# Quality Indicators during Delivery and the Immediate Postpartum Period: A Modified Delphi Study

**DOI:** 10.3390/healthcare11060848

**Published:** 2023-03-13

**Authors:** Candy Guiguet-Auclair, Olivier Rivière, Laurent Gerbaud, Françoise Vendittelli

**Affiliations:** 1 CHU Clermont-Ferrand, CNRS, Institut Pascal, Université Clermont Auvergne, F-63000 Clermont-Ferrand, France; cauclair@chu-clermontferrand.fr (C.G.-A.); lgerbaud@chu-clermontferrand.fr (L.G.); 2 Service de Santé Publique, CHU Clermont-Ferrand, F-63000 Clermont-Ferrand, France; 3 Audipog, Université Claude Bernard Lyon 1-Laennec, 7 Rue Guillaume Paradin, CEDEX 08, F-69372 Lyon, France; olivier.riviere@audipog.net; 4 Réseau de Santé Périnatale d’Auvergne, CHU Clermont-Ferrand, F-63000 Clermont-Ferrand, France

**Keywords:** consensus, Delphi study, maternity care, perinatal health, quality indicators

## Abstract

Background: Our research hypothesis was that most French indicators of quality of care have been validated by experts who are not clinicians and might not always be meaningful for clinicians. Our objective was to define a core set of measurable indicators of care quality during delivery and the immediate postpartum period relevant to clinical practice. Methods: A steering committee comprising nine specialists in obstetrics and/or public health conducted a literature review to develop potential indicators. A panel of obstetrician-gynecologists and midwives working in a delivery unit rated each indicator for appropriateness in a two-round Rand-modified Delphi procedure and a physical meeting. The consensus among the panelists was assessed. Results: In the first round, 145 panelists (110 obstetrician-gynecologists and 35 midwives) assessed 77 indicators and 3 definitions: 6 related to labor onset, 20 to delivery, 3 to pain management, 23 to neonatal morbidity/mortality, and 28 to maternal morbidity. In the second round, 132 panelists (98 obstetrician-gynecologists and 34 midwives) assessed 42 indicators and 1 definition. The final set comprised 50 indicators and 2 definitions. Conclusions: This Delphi procedure selected 50 indicators that reflect the quality of perinatal care. These indicators should be recorded in each French maternity ward’s birth register for each delivery.

## 1. Introduction

Reducing maternal and perinatal severe morbidity and mortality around the world remains a major public health concern [1]. Accordingly, different indicators assessing the quality of perinatal care have been proposed [2,3,4,5,6,7,8,9,10,11,12], although their promulgation and distribution have not resulted in a notable reduction in perinatal and neonatal outcomes around the world [13,14,15,16], or even only in France [17,18,19,20]. These findings, especially given that many are regularly reported to perinatal professionals [19,20,21,22], suggest that these indicators remain less than optimal in France. There are always, of course, variations between countries, between maternity departments within a country, and even between professionals within the same maternity unit [22,23]. One explanation may be that most current maternity indicators derived from routinely collected hospital data are not always easy to understand. This is due mainly to the lack of clinical information contained in these medical-administrative databases and the absence of evidence for the best practice in specific obstetric situations [18,24]. Moreover, indicators derived from routine hospital data can lack validity. For example, “good” maternity wards are considered to be those with a global cesarean rate near the mean for the country. However, what is a good cesarean rate? We do not know; maternity units with low cesarean rates may have high neonatal morbidity and/or mortality, perhaps because their cesarean rates are too low [25]. Several French obstetric guidelines do not provide indicators to assess their impact on the practice or appropriate outcome indicators [26]. Worse, some French national guidelines do not clearly identify the indicators they are intended to affect. For example, the French guidelines about planned cesarean deliveries at term do not define a planned cesarean [27]. 

Our research hypothesis was that as most of the indicators currently used were developed by working groups of experts in quality, public health, or epidemiology, they might not always make sense to clinicians working in delivery rooms. However, it is important to have quality indicators that are well accepted by professionals working in maternity units and are easy to collect so that they can be recorded continuously and enable valid comparisons over time. The National College of French Gynecologists and Obstetricians (CNGOF) and the National College of Midwives (CNSF) have therefore decided to study the opinions of French clinicians practicing in the birth sector about the relevance of quality indicators in maternity units.

Our objective was to use a modified Delphi study to define a core set of quality indicators, measurable and relevant to clinical practice, during delivery and the immediate postpartum period. This technique is widely used to select quality indicators in health care [28].

## 2. Materials and Methods

### 2.1. Design and Setting

**Figure 1:**
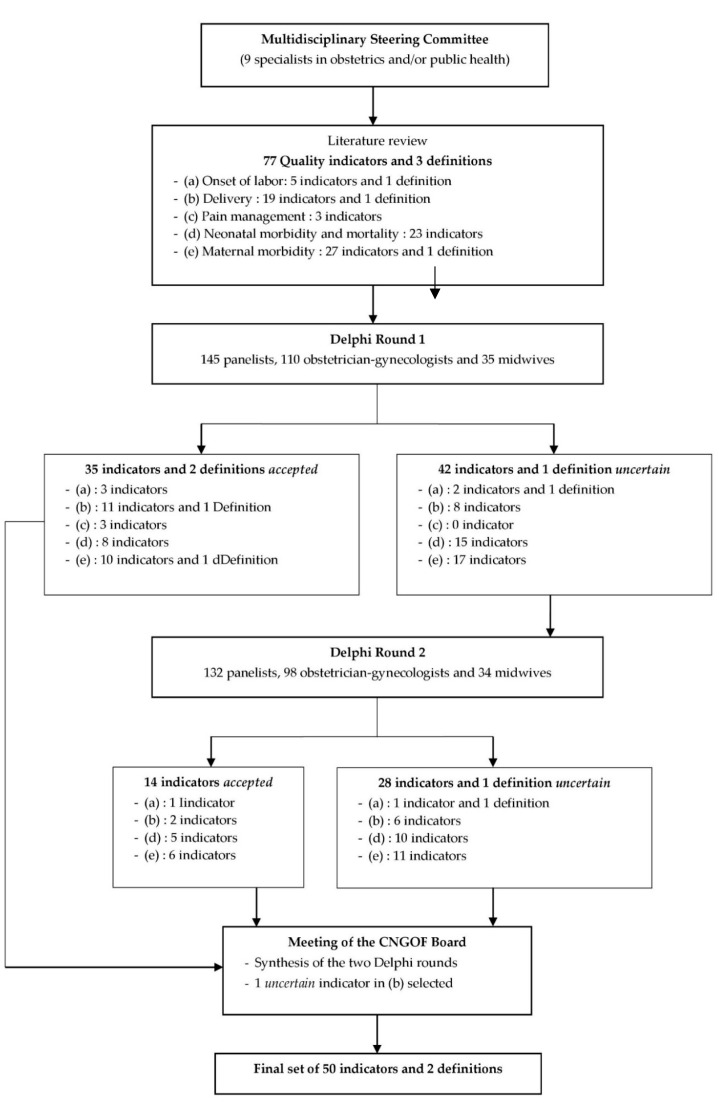
The modified Delphi process for the selection of indicators of quality of care during delivery and the immediate postpartum period.

A modified RAND/University of California at Los Angeles (UCLA) appropriateness method Delphi study took place in four stages (Figure 1) to develop a set of indicators of the quality of care during delivery and the immediate postpartum period for deliveries or births ≥22 weeks (or birth weight ≥ 500 g when the date of conception was unknown) [29]. The first stage was the constitution of a French multidisciplinary steering committee, and the second was the extensive review of the literature it was assigned to perform to identify quality indicators in obstetrics. The third phase consisted of two Delphi consensus rounds in which potential indicators were rated by a panel of informed persons. Delphi is a formalized technique for determining consensus from the collective wisdom of the panelists about, in this context, appropriate indicators. The benefits of this approach are anonymity, iteration (allowing participants to change their opinions), not requiring the physical presence of the panelist, controlled feedback in which panelists can have the panel’s previous responses, and the derivation of summary measures of agreement [30]. In the fourth and last phase, the steering committee and an external board approved the final set of quality indicators. 

We modified the RAND/UCLA appropriateness method by beginning the process with a set of selected quality indicators. The French panelists did not meet physically, and indicators could be discarded between the two rounds. 

### 2.2. Literature Review

The French steering committee established comprised nine specialists in obstetrics and/or public health, recognized for their expertise in quality indicators and/or the Delphi method. This committee reviewed the literature from January 2003 through December 2013. A PubMed search was conducted for the keywords “quality indicators” and “obstetrics”. All reports on perinatal quality indicators issued by learned societies in France or abroad were also collected. A database of 318 references was identified. 

During face-to-face and telephone meetings, completed by email exchanges, the steering committee used the literature search results to establish an exhaustive list of potential quality indicators. The indicators to be chosen were to be collected and calculated from data in patient files, collected immediately intrapartum, during delivery, and early postpartum (in the delivery or operating unit, according to the type of delivery). 

The Delphi questionnaire was then drafted, with special attention paid to the clarity of each indicator. The web-based questionnaire and instructions were tested before each round for face validity. 

### 2.3. Modified Delphi Process

#### 2.3.1. Participants

The list of quality of care indicators was presented for rating in a two-round Delphi study. Obstetrician-gynecologists who were members of CNGOF and midwives belonging to the CNSF—all practicing in French delivery rooms—were invited via each organization’s email list to compose the panel of the Delphi study. They received a cover letter by email explaining the study and inviting them to participate by completing the electronic questionnaire accessible with the URL in the email. No financial incentive was proposed. The panelists gave their consent to participate in the Delphi survey when they replied to the first round. Under French law, this study was exempt from approval by an ethics committee.

#### 2.3.2. Data Collection

Round 1 data were collected from 11 December 2014 to 28 January 2015, and round 2 data from 25 March to 17 May 2015. The participants were asked to answer within four weeks for each round. No reminder was sent out during the first round, whereas two reminder emails were sent during the second. Only participants who completed the first-round electronic questionnaire received the URL to participate in the second round. 

During both rounds, the panelists were invited to rate individually and independently each quality indicator for its clinical relevance on a 9-point Likert scale from 1 (totally inappropriate/irrelevant) to 9 (totally appropriate/relevant), with 5 for no preference, indecision, etc. They were also invited to comment on each indicator.

For the second round, the panelists received descriptive statistics of the distribution of the panel’s scores for each indicator rated in the first round. Each panelist was invited to re-score on the same 9-point Likert scale the indicators that had not been accepted in the first round, taking into account the previous answers of the panel.

#### 2.3.3. Data Analysis 

After each of the two rounds, the distribution of scores was described by the number and frequencies of each score of the rating scale, the number and frequencies in the lowest tertile (between 1 and 3), the number and frequencies in the upper tertile (between 7 and 9), and the median score.

Judgment of the indicators and consensus followed the RAND/UCLA method [29]. A median score was used to judge an indicator by measuring the central tendency of the distribution for each indicator’s rating. An indicator was judged *appropriate* if the panelists’ median score ranged from 7 through 9. An indicator with a median score of 1–3 was judged *inappropriate*. To evaluate the consensus between the panelists (that is, the panelists’ agreement with one another), a continuous statistical measure of dispersion among the individual scores was used: the Disagreement Index. We adapted the Rand Working Group definition and defined the Disagreement Index as the 10–90% interpercentile range (IPR) divided by the interpercentile range adjusted for symmetry (IPRAS) [29], which applies to any size panel. In the RAND method, a Disagreement Index lower than 1 indicates consensus or agreement between panelists (low dispersion of scores, with the IPRAS larger than the IPR), and a Disagreement Index higher than 1 indicates a lack of consensus or disagreement (high score dispersion with the IPRAS smaller than the IPR). 

If an indicator was judged *appropriate* with agreement among the panelists, then it was considered *accepted*. Indicators consensually judged *inappropriate* were *rejected*. An indicator with a median score ranging between 3.5 and 6.5 or scored with disagreement between panelists was considered *uncertain*. Based on the findings of round 1, *uncertain* indicators were resubmitted for further evaluation and discussion in the second rating round. 

Statistical analyses were performed with SAS software (version 9.3, SAS Institute, Cary, NC, 2002–2012).

### 2.4. External Validity 

The steering committee received the detailed results after each round and discussed them. In November 2015, after the second round, the Delphi results were sent for validation to the CNGOF and CNSF board members. The final set of quality indicators was established.

## 3. Results

### 3.1. Modified Delphi Survey Questionnaire

The steering committee proposed a total of 77 indicators and 3 definitions for evaluation in the two Delphi rounds, categorized as follows: “onset of labor” (5 indicators and 1 definition), “delivery” (19 indicators and 1 definition), “pain management” (3 indicators), “neonatal morbidity and mortality” (23 indicators), and “maternal morbidity” (27 indicators and 1 definition) (Figure 1). Indicator results were expressed as percentages. Numerators (number of the exposed population by the relevant event) and denominators (target population) were detailed for each quality indicator. 

### 3.2. Description of the Modified Delphi Survey Panel

The first round was completed by 145 panelists, 110 obstetrician-gynecologists (75.9%) practicing for 20.5 ± 10.1 years, and 35 midwives (24.1%), practicing for 21.7 ± 11.6 years (Table 1). Men accounted for 54.5%, and the mean age of the entire panel was 49.7 ± 10.6 years. Overall, 34.5% practiced in academic hospitals, 41.4% in general public hospitals, and 24.1% in private hospitals; 20.7% worked in level I maternity units, 40.7% in level II facilities, and 38.6% in level III hospitals, with 50.3% practicing in a maternity unit with an average of more than 2500 deliveries annually. 

Participating in the second round were 132 panelists who had responded to the first round—98 obstetrician-gynecologists and 34 midwives (89.1% and 97.1%, respectively) (Table 1). They had been in practice for a mean of 21.5 ± 10.6 years, and 53.0% practiced in a maternity unit with a mean of more than 2500 annual deliveries. 

### 3.3. Modified Delphi Survey Analysis

Figure 1 presents the modified Delphi process used to select the quality indicators.

After the first round, 35 indicators and 2 definitions were *accepted*: 3 indicators in the domain “onset of labor”, 11 indicators and 1 definition in “delivery”, all the indicators in “pain management” (i.e., 3), 8 indicators in “neonatal morbidity and mortality”, and 10 indicators and 1 definition in “maternal morbidity”. No indicator was *rejected*. Forty-two indicators and 1 definition were considered *uncertain* and proposed for further evaluation in the second round (Table 2). 

After the second round, 14 indicators were *accepted*: 1 indicator in the “onset of labor” domain, 2 in “delivery”, 5 in “neonatal morbidity and mortality”, and 6 in “maternal morbidity”. No indicator was *rejected,* while 28 indicators and 1 definition remained *uncertain* (Table 2). The indicators (including their definitions) that remained *uncertain* after the two rounds are listed in Supplementary Table S1. 

### 3.4. External Validity

A synthesis of the two rounds of the Delphi process was presented and discussed in a face-to-face meeting of the CNGOF board members. Among the indicators that had not been accepted, one was judged highly important based on the international literature review and was included in the final set of quality indicators: the Robson classification [31], advocated by the International Federation of Gynecology and Obstetrics (FIGO) [32]. 

Finally, a set of 50 quality indicators and 2 definitions was established: 4 indicators in the domain of “onset of labor”, 15 indicators and 1 definition in “delivery”, 3 indicators in “pain management”, 13 indicators in “neonatal morbidity and mortality”, and 16 indicators and 1 definition in “maternal morbidity”. Table 3 presents the detailed definition (numerator and denominator) for each final quality indicator. 

## 4. Discussion

Using a modified RAND-UCLA Delphi method, we developed a clinically relevant set of 50 quality indicators and 2 definitions in obstetrics that covered care during delivery and in the immediate postpartum period. They were based simultaneously on medical literature and on the judgments of a large panel. One indicator—the Robson classification for cesareans—was selected after the Delphi process by the CNGOF in view of international guidelines and to promote international comparability by the FIGO [32]. 

The number of panelists in the two-round Delphi survey is one of the strengths of our study. To the best of our knowledge, there are currently no clear guidelines for the sample size of a Delphi panel [33]. It has been suggested that a minimum number of panelists should range from 7 to 15 [29,34]. The large panel of obstetrician-gynecologists and midwives who participated in this Delphi survey should certainly have increased the stability of our results and the reliability of the final set of quality indicators [35,36]. Second, only a few panelists did not respond to the second round of Delphi, and the follow-up response rate exceeded the 70% suggested by Sumsion [37]. Another strength of our study was the assessment of external validity. Indeed, the final set of quality indicators was submitted for an external independent review for approval by two French professional societies specialized in childbirth: CNGOF and CNSF—whose members are the obstetricians and midwives who complete the delivery register in the maternity units for each delivery. Similarly, the only eligible panelists were obstetricians and midwives working in a delivery unit. Our final objective was to select indicators that can be routinely filled in and monitored via the computerization of the delivery registers available in all French maternity units to guide the development of quality improvement programs at the local and national levels.

This study has some limitations. The indicators were developed in a French setting, so the results may not be generalizable to other countries as practices vary internationally. Nonetheless, the recommendations were based on references extracted from an international literature review, which should provide good external validity. The self-selection of the panelists may have influenced the results. Those who participated in the Delphi rounds may not have had the same characteristics and/or may not have rated the indicators the same as those who did not participate. To facilitate a high level of continued participation, the number of rounds for the Delphi procedure was limited to 2. This left some indicators or definitions neither accepted nor rejected. This is the case for the definition of a planned cesarean, which remains a problem in France. We have guidelines about planned cesareans and a national indicator (rate of cesareans [%]) but still no clear definition of a planned cesarean [27,38].

Globally, the panelists did not select any of the risk-adjusted indicators or adverse outcome index models suggested by various authors [12,39,40,41]. However, they did select the main’s cesarean indicator “nulliparous term singleton vertex cesarean birth” (NTSV CB) [42]. This underlines the incomprehensibility of risk-adjusted indicators for clinicians without substantial training and experience in statistics.

During the first Delphi round, the panelists accepted the international definitions about perineal lacerations, which will enable comparisons of French studies with non-French studies and quality indicators on this topic. To our surprise, the definition of low risk at the end of pregnancy was accepted during the first Delphi round, although there was no formal consensus in France on this subject at that time.

We expected a lower number of quality indicators to be selected after the second round because mandatory care quality and safety indicators in French hospitals are poorly accepted by caregivers [43]. The results of these indicators contribute to the pay-for-performance mechanism of French health establishments [44]. However, the bulk of hospital revenues is still allocated according to medical activities. This medical resistance to guideline implementation, assessment by indicators, and audits point to the physicians’ feeling of loss of autonomy through the demands of standardization of medical practices [45,46,47]. We were therefore surprised by the high number of perinatal care providers participating in the Delphi procedure.

For many indicators, we do not know what the right rate is [48]. For example, the planned cesarean rate is a good indicator of the quality of care in a maternity ward. Nonetheless, it is difficult to tell physicians what the correct rate of this intervention is. The mean rate is usually recommended, on the assumption that the highest and lowest rates are inappropriate, but this is not necessarily true. In a study, we assessed the incidence of postpartum hemorrhage (PPH) and second-line procedures in maternity units according to the quality of their PPH protocol [49]. We find that maternity units with higher scores identified PPH better and used fewer curative second-line procedures. So the ideal rate is that associated with the lowest level of maternal and/or neonatal morbidity or mortality, but it is difficult to ascertain in practice.

The number of indicators selected at the end of our study is reduced compared to some reviews of the literature [50,51]. This can be explained by the fact that we have focused on indicators that can be recorded in the daily routine in the birth unit and so we do not have structure or health-determinant quality indicators [50].

## 5. Conclusions

In conclusion, our study has identified a list of 50 quality indicators and 2 definitions suitable for routine monitoring in maternity units. However, the feasibility of these quality indicators will need to be assessed by research studies conducted under the conditions of everyday practices. To improve the calculation and monitoring of these indicators, birth registers, still widely kept on paper in French maternity wards, should be required to be computerized. Further research should study the impact of monitoring these indicators on improving maternal and neonatal morbidity and mortality at a national level.

## Figures and Tables

**Table 1:** Panelists’ characteristics.

Rounds of Modified Delphi Survey	Variables	Obstetrician-Gynecologists	Midwives	Total
First Round	N of participants	110	35	145
	Age (years), mean ± SD	51.5 ± 9.6	45.4 ± 11.6	49.7 ± 10.6
	Sex, n (%)			
	Female	32 (29.1)	34 (97.1)	66 (45.5)
	Male	78 (70.9)	1 (2.9)	79 (54.5)
	Professional experience (years), n (%)			
	<10	19 (17.3)	8 (22.9)	27 (18.6)
	≥10–<20	30 (27.3)	4 (11.4)	34 (23.4)
	≥20–<30	37 (33.6)	12 (34.3)	49 (33.8)
	≥30	24 (21.8)	11 (31.4)	35 (24.1)
	Current place of work ^1^, n (%)			
	Academic hospital	37 (33.6)	13 (37.1)	50 (34.5)
	General public hospital	43 (39.1)	17 (48.6)	60 (41.4)
	Private hospital	30 (27.3)	5 (14.3)	35 (24.1))
	Maternity unit level ^2^, n (%)			
	Level I	27 (24.5)	3 (8.6)	30 (20.7)
	Level II	41 (37.3)	18 (51.4)	59 (40.7)
	Level III	42 (38.2)	14 (40.0)	56 (38.6)
	Mean number of annual deliveries, n (%)			
	<500	3 (2.7)	0	3 (2.1)
	≥500–<1500	29 (26.4)	10 (28.6)	39 (26.9)
	≥1500	78 (70.9)	25 (71.4)	103 (71.0)
Second Round	N of participants	98	34	132
	Age (years), mean ± SD	51.5 ± 9.6	46.3 ± 11.7	50.2 ± 10.4
	Sex, n (%)			
	Female	29 (29.6)	1 (2.9)	62 (47.0)
	Male	69 (70.4)	33 (97.1)	70 (53.0)
	Professional experience (years), n (%)			
	<10	17 (17.3)	7 (20.6)	24 (18.2)
	≥10–<20	27 (27.6)	5 (14.7)	32 (24.2)
	≥20–<20	31 (31.6)	10 (29.4)	41 (31.1)
	≥30	23 (23.5)	12 (35.3)	35 (26.5)
	Current place of work ^1^, n (%)			
	Academic hospital	35 (35.7)	13 (38.2)	48 (36.4)
	General public hospital	37 (37.8)	16 (47.1)	53 (40.2)
	Private hospital	26 (26.5)	5 (14.7)	31 (23.5)
	Maternity unit level ^2^, n (%)			
	Level I	21 (21.4)	3 (8.8)	24 (18.2)
	Level II	37 (37.8)	17 (50.0)	54 (40.9)
	Level III	40 (40.8)	14 (41.2)	54 (40.9)
	Mean number of annual deliveries, n (%)			
	<500	1 (1.0)	0	1 (0.8)
	≥500–<1500	25 (25.5)	10 (29.4)	35 (26.5)
	≥1500	72 (73.5)	24 (70.6)	96 (72.7)

^1^ Academic hospital: regional public hospital connected with a university in France; general public hospital: not connected with a university; private hospital: in the private sector, whether for profit or not for profit. ^2^ Level I: without a neonatology department; Level II: with a neonatology department; Level III: with a neonatology department and a neonatal intensive care unit.

**Table 2:** Rating scores of the proposed quality indicators and definitions during the two-round Delphi survey.

Category	Quality Indicators ^1^ or Definition	First Round (*n* = 145)			Second Round ^2^ (*n* = 132)		
		Median Score ^3^	Disagreement Index	Accepted, Rejected, or Uncertain ^4^	Median Score ^3^	Disagreement Index	Accepted, Rejected, or Uncertain ^4^
Onset of labor	Spontaneous labor	8	2.3	Uncertain	8	1.6	Uncertain
	Inductions of labor	8	0.7	Accepted			
	Planned cesarean deliveries	9	0.5	Accepted			
	Planned cesareans ≥ 39 wk	8	0.7	Accepted			
	Definition of a planned cesarean delivery	8	1.6	Uncertain	8	1.6	Uncertain
	Cesarean deliveries before labor	8	1.1	Uncertain	8	0.7	Accepted
Delivery	Vaginal deliveries	9	0.3	Accepted			
	Spontaneous vaginal deliveries (non-operative)	9	0.5	Accepted			
	Instrumental vaginal deliveries	9	0.3	Accepted			
	Global cesarean deliveries	9	0.3	Accepted			
	Global cesarean deliveries ≥ 37 wk with a singleton pregnancy and a breech presentation	9	0.7	Accepted			
	Cesarean deliveries in nulliparas	9	0.7	Accepted			
	Cesareans in primiparas with one previous cesarean ^5^	8	2.3	Uncertain	8	2.3	Uncertain
	Cesarean deliveries during labor	8	0.7	Accepted			
	Adjusted proportion of cesarean deliveries	7	1.1	Uncertain	7	1.1	Uncertain
	Cesareans ≥37 wk in nulliparas women with a singleton pregnancy and a cephalic presentation	9	1.1	Uncertain	9	0.7	Accepted
	Cesarean deliveries after one previous cesarean	9	0.5	Accepted			
	Cesarean deliveries in twin pregnancy	9	0.5	Accepted			
	Planned cesarean deliveries ≥ 39 wk	8	0.7	Accepted			
	Cesarean deliveries < 32 wk	8	2.3	Uncertain	8	1.6	Uncertain
	Unplanned cesareans after failure of labor induction	9	0.7	Accepted			
	Unplanned cesareans after spontaneous onset of labor	8	1.6	Uncertain	8	1.6	Uncertain
	Definition of low risk at the end of pregnancy	8	0.7	Accepted			
	Cesarean deliveries in women at low risk	8	1.6	Uncertain	8	0.7	Accepted
	Maternity Unit Performance Index	5	4.4	Uncertain	5	4.4	Uncertain
	Cesarean deliveries according to Robson classification	6	1.6	Uncertain	7	1.6	Uncertain
Pain management	General anesthesia for cesarean delivery	9	0.7	Accepted			
	Epidural analgesia for vaginal delivery	9	0.3	Accepted			
	Spinal analgesia for cesarean delivery	9	0.7	Accepted			
Neonatal morbidity and mortality	Apgar score < 7 at 5 min	8	1.1	Uncertain	8	0.7	Accepted
	Apgar score < 5 at 5 min	8	1.1	Uncertain	8	1.6	Uncertain
	Births ≥37 wk with Apgar score <7 at 5 min	8	0.7	Accepted			
	Live-born preterm infants < 37 wk	9	0.7	Accepted			
	Live-born infants, preterm birth < 34 wk	8	0.7	Accepted			
	Live-born infants, preterm birth < 33 wk	7	3.4	Uncertain	7	3.4	Uncertain
	Live-born infants, preterm birth < 32 wk	8	1.1	Uncertain	8	0.7	Accepted
	Live-born infants, preterm birth < 28 wk	9	0.7	Accepted			
	Arterial pH < 7.10	8	1.6	Uncertain	8	0.7	Accepted
	Arterial pH < 7.0	8	1.1	Uncertain	8	0.5	Accepted
	Birth weight < 5th percentile for gestational age	8	1.6	Uncertain	8	1.6	Uncertain
	Birth weight < 3rd percentile for gestational age	8	0.7	Accepted			
	Birth weight < 2750 g with birth ≥ 40 wk	6	3.4	Uncertain	6	3.4	Uncertain
	Birth weight < 2500 g	7	3.4	Uncertain	7	3.4	Uncertain
	Infant discharged from delivery unit with mother and birth ≥ 37 wk	8	2.3	Uncertain	8	1.6	Uncertain
	Neonatal transfers in resuscitation or intensive care or neonatology unit	9	0.7	Accepted			
	Neonatal transfers in intensive care unit	9	1.1	Uncertain	9	0.7	Accepted
	Neonatal transfers in intensive care unit with birth weight > 2500 g	7	1.6	Uncertain	7	1.6	Uncertain
	Neonatal transfers to any neonatal units for inborn infants with birth ≥ 37 wk	9	0.7	Accepted			
	Neonatal transfers to any neonatal units for inborn infants with birth ≥ 22 wk	7	2.3	Uncertain	7	3.4	Uncertain
	Uncomplicated births with a healthy infant	8	1.1	Uncertain	8	1.6	Uncertain
	Stillbirths	9	0.7	Accepted			
	Stillbirths ≥ 28 wk	8	1.1	Uncertain	8	1.6	Uncertain
Maternal morbidity	Postpartum hemorrhages > 500 mL (regardless of mode of delivery)	9	1.6	Uncertain	9	1.6	Uncertain
	Postpartum hemorrhages > 500 mL for vaginal delivery	9	0.7	Accepted			
	Postpartum hemorrhages > 1000 mL (regardless of mode of delivery)	9	1.1	Uncertain	9	0.7	Accepted
	Postpartum hemorrhages > 1000 mL for vaginal delivery	9	1.1	Uncertain	9	0.7	Accepted
	Postpartum hemorrhages > 1000 mL for cesarean delivery	9	0.7	Accepted			
	Postpartum hemorrhages > 1500 mL (regardless of mode of delivery)	8	2.3	Uncertain	8	2.3	Uncertain
	Postpartum hemorrhages > 1500 mL for cesareans	7	2.3	Uncertain	8	2.3	Uncertain
	Blood transfusions in delivery or operating rooms	9	0.5	Accepted			
	Hemostatic hysterectomy	9	0.3	Accepted			
	Hemostatic surgery (other than hysterectomy)	9	0.3	Accepted			
	Uterine rupture	9	1.1	Uncertain	9	0.7	Accepted
	Discharge from delivery unit by transfer to intensive care unit	9	0.7	Accepted			
Maternal morbidity	Intact perineum (without perineal suture)	9	0.7	Accepted			
	Intact perineum in spontaneous delivery	8	3.4	Uncertain	8	1.6	Uncertain
	Intact perineum in primiparas	8	2.3	Uncertain	8	2.3	Uncertain
	Episiotomies	9	0.3	Accepted			
	Episiotomies in primiparas	8	1.6	Uncertain	8	0.5	Accepted
	Perineal lacerations (perineal tears and/or episiotomy)	8	1.6	Uncertain	8	3.4	Uncertain
	Perineal lacerations without episiotomy	8	1.6	Uncertain	8	1.6	Uncertain
	First-degree perineal tears ^6^	7	3.4	Uncertain	7	3.4	Uncertain
	Second-degree perineal tears ^7^	7	2.3	Uncertain	8	2.3	Uncertain
	Severe perineal tears (3rd- or 4th-degree) ^8^	9	0.3	Accepted			
	Severe perineal tears in instrumental vaginal delivery	9	0.7	Accepted			
	Severe perineal tears in non-instrumental vaginal delivery	9	1.1	Uncertain	9	0.3	Accepted
	Severe perineal tears in forceps delivery	8	2.3	Uncertain	8	0.7	Accepted
	Severe perineal tears in vacuum delivery	8	2.3	Uncertain	8	1.6	Uncertain
	Adjusted proportion of severe perineal lacerations	8	3.4	Uncertain	8	3.4	Uncertain
	International definition of perineal tears ^6,7,8^ (first, second, third, and fourth degree)	9	0.3	Accepted			

Wk: weeks of amenorrhea. ^1^ Quality indicators are expressed as percentages. ^2^ Quality indicators or definitions *uncertain* in the first round were resubmitted. Quality indicators and definitions *accepted* in the first round were not included in the second round. ^3^ Each quality indicator or definition was rated on a 9-point Likert scale where 1 = totally inappropriate/irrelevant and 9 = totally appropriate/relevant. ^4^ A quality indicator or definition consensually judged appropriate (median score of 7–9 and Disagreement Index < 1) was *accepted*. A quality indicator or definition consensually judged inappropriate (median score of 1–3 and Disagreement Index < 1) was *rejected*. A quality indicator or definition with a median score of 3.5–6.5 or scored nonconsensually (Disagreement Index > 1) was considered *uncertain* and reconsidered in the second round. ^5^ Women with a history of only one child born by cesarean and who have a secondary cesarean for their second delivery. ^6^ First-degree tear involves damage to vaginal and perineal skin. ^7^ Second-degree tear involves the posterior vaginal wall and the underlying elevator and perineal muscles. ^8^ Severe perineal tears include 3rd and 4th degree perineal tears. Third-degree tears involve the anal sphincter, with either total or partial damage to the sphincter, and fourth-degree tears involve the anal sphincter and tears into the rectal mucosa.

**Table 3:** Final set of quality indicators and definitions.

Category	Quality Indicators ^1^ or Definitions	Numerator	Denominator
**Onset of labor**	Inductions of labor	Number of inductions of labor ^2^	Total number of deliveries ^2^
	Planned cesarean deliveries	Number of planned cesarean deliveries performed on or before the scheduled due date (surgical indication recorded ≥ 48 h before performance) ^2^	Total number of cesarean deliveries ^2^
	Planned cesareans ≥ 39 wk	Number of planned cesarean deliveries ≥ 39 wk performed on or before the scheduled due date (surgical indication recorded ≥ 48 h before performance) ^2^	Total number of planned cesarean deliveries ^2^
	Cesareans before labor	Number of cesarean deliveries before labor ^2^	Total number of cesarean deliveries ^2^
**Delivery**	Vaginal deliveries	Number of vaginal deliveries ^2^	Total number of deliveries ^2^
	Spontaneous vaginal deliveries (non-instrumental)	Number of non-instrumental spontaneous vaginal deliveries ^2^	Total number of vaginal deliveries ^2^
	Instrumental vaginal deliveries	Number of instrumental vaginal deliveries ^2,3^	Total number of vaginal deliveries ^2^
	Global cesarean deliveries	Number of cesareans ^2^	Total number of deliveries ^2^
	Global cesareans ≥ 37 wk with a singletonpregnancy in breech presentation	Number of cesareans ≥ 37 wk with a singleton pregnancy in breech presentation ^2^	Total number of deliveries ≥ 37 wk with a singleton pregnancy in breech presentation ^2^
	Cesareans among nulliparas	Number of cesareans among nulliparas ^2^	Total number of deliveries for nulliparas ^2^
	Cesareans during labor	Number of cesareans during labor (among planned cesareans or planned vaginal deliveries) ^2^	Total number of cesarean deliveries ^2^
	Cesareans ≥ 37 wk for nulliparas with a singleton pregnancy in cephalic presentation	Number of cesareans ≥ 37 wk for nulliparas with a singleton pregnancy in cephalic presentation ^2^	Total number of deliveries ≥ 37 wk for nulliparas with a singleton pregnancy in cephalic presentation ^2^
	Cesareans after one previous cesarean	Number of cesareans after only one previous cesarean delivery ^2^	Total number of deliveries with only oneprevious cesarean delivery ^2^
	Cesareans in non-singleton pregnancy	Number of cesareans in non-singleton pregnancy ^2^	Total number of non-singleton deliveries ^2^
	Planned cesareans ≥ 39 wk	Number of planned cesareans ≥ 39 wk ^2^	Total number of cesarean deliveries ^2^
	Unplanned cesareans after failure of induction	Number of unplanned cesareans after induction of labor ^2^	Total number of inductions of labor ^2^
	Definition of low risk at the endof pregnancy	Low-risk women at the end of pregnancy defined as initially at low risk at the beginning of pregnancy (1), and at low risk during pregnancy (2), without placenta previa or other obstacle for a vaginal delivery, with a fetus in cephalic presentation.	
		(1) Initially (at first consultation) at low risk: - Age: ≥18 years and <35 years - No medical history negatively affecting the pregnancy - No history of preterm birth, stillbirth, neonatal death - Singleton pregnancy - No previous cesarean or other uterine scar	(2) Low-risk women during pregnancy: - Initially at low risk and no disorders of pregnancy - No threatened premature delivery - No hypertension - No diabetes - No amniotic fluid abnormality - No uterine growth abnormality
	Cesareans in women at low risk	Number of cesareans in women at low risk ^2,4^	Total number of deliveries in women at low risk ^2^
	Cesarean sections according to Robson 10-group classification	Number of cesareans ^2^ in: Nulliparas, singleton cephalic fetus, ≥37 wk, in spontaneous labor Nulliparas, singleton cephalic fetus ≥37 wk, induction or cesarean before labor Multiparas (excluding previous cesarean), singleton cephalic fetus, ≥37 wk, in spontaneous labor Multiparas (excluding previous cesarean), singleton cephalic fetus, ≥37 wk, induction or cesarean before labor Previous cesarean, singleton cephalic fetus, ≥37 wk All nulliparas with fetus in breech presentation All multiparas with fetus in breech presentation (including previous cesarean) All multiple pregnancies (including previous cesarean) All abnormal lies (including previous cesarean) All singleton cephalic fetuses, ≤36 wk (including previous cesarean)	Total number of deliveries ^2^ according to the description of each group (for example, for group 1: total number of deliveries in nulliparas, with a singleton cephalic fetus, ≥37 wk, in spontaneous labor)
**Pain management**	General anesthesia for cesarean delivery	Number of women receiving general anesthesia for cesareans ^2^	Total number of cesarean deliveries ^2^
	Epidural analgesia for vaginal delivery	Number of women receiving epidural analgesia for vaginal delivery ^2^	Total number of vaginal deliveries ^2^
	Spinal analgesia for cesareans	Number of women receiving spinal analgesia for cesareans ^2^	Total number of cesarean deliveries ^2^
**Neonatal morbidity and mortality**	Apgar score < 7 at 5 min	Number of live-born infants with Apgar score < 7 at 5 min ^2^	Total number of live-born infants (stillborn infants excluded) ^2^
	Births ≥ 37 wk with Apgar score < 7 at 5 min	Number of live-born infants with a birth ≥ 37 wk and an Apgar score < 7 at 5 min ^2^	Total number of live-born infants ≥ 37 wk (stillborn infants excluded) ^2^
	Live-born preterm neonate	Number of live-born infants before < 37 wk gestation ^2^	Total number of live-born infants ^2^
	Live-born infants before < 34 wk gestation	Number of live-born infants before < 34 wk gestation ^2^	Total number of live-born infants ^2^
	Live-born infants before < 32 wk gestation	Number of live-born infants before < 32 wk gestation ^2^	Total number of live-born infants ^2^
	Live-born infants before < 28 wk gestation	Number of live-born infants before < 28 wk gestation ^2^	Total number of live-born infants ^2^
	Arterial pH < 7.10	Number of live-born infants with umbilical artery pH < 7.10 ^2^	Total number of live-born infants with umbilical artery pH measured ^2^
	Arterial pH < 7.0	Number of live-born infants with umbilical artery pH < 7.0 ^2^	Total number of live-born infants with umbilical artery pH measured ^2^
	Birth weights < 3rd percentile for gestational age	Number of infants with a birth weight < 3rd percentile for gestational age ^2^	Total number of newborns ^2^
	Neonatal transfers to intensive care or neonatology unit	Number of live-born infants transferred to an intensive care or neonatology unit ^2^	Total number of live-born infants (admissions for congenital malformations and transfer to kangaroo care unit excluded) ^2^
	Neonatal transfers to intensive care unit	Number of live-born infants transferred to intensive care unit ^2^	Total number of live-born infants (admissions for congenital malformations excluded) ^2^
	Immediate transfers to any neonatal unit for infants born ≥ 37 wk	Number of infants born ≥37 wk transferred to any neonatal unit ^2^	Total number of live-born infants ≥ 37 wk (admissions for congenital malformations excluded) ^2^
	Stillbirths	Number of fetal or immediate neonatal deaths (including fetal deaths in utero, medical terminations of pregnancy, and intrapartum deaths) ^2^	Total number of births (live-born or stillborn infants) ^2^
**Maternal morbidity**	Postpartum hemorrhages >500 mL for vaginal delivery	Number of postpartum hemorrhages > 500 mL for vaginal delivery ^2^	Total number of vaginal deliveries ^2^
	Postpartum hemorrhages > 1000 mL (regardless of mode of delivery)	Number of postpartum hemorrhages > 1000 mL for delivery ^2^	Total number of deliveries ^2^
	Postpartum hemorrhage > 1000 mL for vaginal delivery	Number of postpartum hemorrhages > 1000 mL for vaginal delivery ^2^	Total number of vaginal deliveries ^2^
	Postpartum hemorrhages > 1000 mL for cesarean deliveries	Number of postpartum hemorrhages > 1000 mL for cesareans ^2^	Total number of cesarean deliveries ^2^
	Blood transfusions in delivery or operating room	Number of blood transfusions in the delivery or operating room ^2^	Total number of deliveries ^2^
	Hemostatic hysterectomies	Number of hemostatic hysterectomies ^2^	Total number of deliveries ^2^
	Hemostatic surgery (other than hysterectomy)	Number of hemostatic surgeries other than hysterectomies ^2^	Total number of deliveries ^2^
	Uterine ruptures	Number of complete or subperitoneal uterine ruptures (dehiscences excluded) ^2^	Total number of deliveries ^2^
	Discharge from delivery unit by transfer to adult intensive care unit	Number of women discharged from the delivery unit by a transfer to an intensive care unit ^2^	Total number of deliveries ^2^
	Intact perineum	Number of women with an intact perineum (without suture) ^2^	Total number of deliveries ^2^
	Episiotomies	Number of women with an episiotomy for vaginal delivery ^2^	Total number of vaginal deliveries ^2^
	Episiotomies in primiparas	Number of primiparas with an episiotomy in vaginal delivery ^2^	Total number of vaginal deliveriesin primiparas ^2^
	Severe perineal tears	Number of women with 3rd- and 4th-degree perineal tears ^5^ in vaginal delivery ^2^	Total number of vaginal deliveries ^2^
	Severe perineal tears for instrumental vaginal delivery	Number of women with 3rd- and 4th-degree perineal tears ^5^ for instrumental vaginal delivery ^2^	Total number of instrumental vaginal deliveries (forceps, vacuum, spatula) ^2^
	Severe perineal tears for non-instrumental vaginal delivery	Number of women with 3rd- and 4th-degree perineal tears ^5^ in non-instrumental vaginal delivery ^2^	Total number of non-instrumental vaginal deliveries ^2^
	Severe perineal tears for forceps delivery	Number of women with 3rd- and 4th-degree perineal tears ^5^ ininstrumental vaginal delivery using forceps ^2^	Total number of instrumental vaginal deliveries using forceps ^2^
	International definition of perineal tears (first, second, third, and fourth degrees)	1st degree: tears affecting the skin or the vaginal mucosa 2nd degree: tears affecting the muscle of the perineum but not the sphincter 3rd degree: tears affecting the anal sphincter 4th degree: tears involving the anal sphincter and tears into the rectal mucosa	

Wk: weeks of amenorrhea. ^1^ Quality indicators are expressed as percentages. ^2^ For births ≥ 22 wk or birth weight ≥ 500 g in the absence of a known pregnancy onset. ^3^ Forceps, spatula, or vacuum extractor. ^4^ According to the previously proposed definition during the Delphi process. ^5^ Third-degree tears involve the anal sphincter, with either total or partial damage to the sphincter and fourth-degree tears involving the anal sphincter and tears into the rectal mucosa.

## Data Availability

The data that support the findings of this study are available from the corresponding author upon reasonable request.

## Supplementary Materials

The following supporting information can be downloaded at: https://www.mdpi.com/article/10.3390/healthcare11060848/s1, Supplementary Table S1: Quality indicators (numerator and denominator) and definitions that were not accepted after both rounds of the Delphi survey.
