# Supplementary material for: Quality Indicators during Delivery and the Immediate Postpartum Period: A Modified Delphi Study
Source: Healthcare (Basel). 2023 Mar 13;11(6):848. doi: 10.3390/healthcare11060848 (PMC10048479; doi:10.3390/healthcare11060848)
Supplement: Supplementary file 1 [file healthcare-11-00848-s001.zip › healthcare-2242168-supplementary.pdf]

Supplementary Table S1. Quality indicators (numerator and denominator) and definitions that were not accepted after both rounds of the Delphi survey.

Category	Quality indicators [1] or Definitions	Numerator	Denominator
Onset of labor	Spontaneous labor	Number of women with spontaneous labor [2]	Total number of deliveries [2]
	Definition of a planned cesarean delivery	Surgical indication of a cesarean recorded ≥48 hours before its performance	
Delivery	Cesareans in primiparas with one previous cesarean [3]	Number of cesareans among primiparas with one previous cesarean [2]	Total number of deliveries among primiparas [2]
	Adjusted proportion of cesarean deliveries	Expected cesarean rate is calculated by adjustment with multivariate statistical methods that take into account women's demographic factors (e.g. age, etc.) and disorders (e.g. hypertension)	
	Cesarean deliveries <32 wk	Number of cesareans <32 wk [2]	Total number of deliveries <32 wk [2]
	Unplanned cesareans after Spontaneous onset of labor	Number of unplanned cesareans after spontaneous labor [2]	Total number of women with spontaneous labor [2]
	Maternity Unit Performance Index	The Maternity Unit Performance Index (MUPI) is calculated by assigning a contributor index of +1, +0.5, −1, −0.5 to various positive and negative obstetric performance parameters. Obstetric components of MUPI and their contributor index assignments are:<br>- vaginal delivery (VD), spontaneous labor (SpL): +1<br>- assisted deliveries (AssD), induction of labor (IOL): +0.5<br>- cesarean delivery (C/D), elective cesarean delivery (elC/D): −1<br>- 3rd and 4th degree perineal tears (TR): −0.5<br>- blood transfusion (BT) required during the first 2 hours postpartum: −0.5<br>- perinatal mortality rate (PMR): −1<br>- maternal mortality rate (MMR): −1.<br>The MUPI formula is the following:<br>MUPI=VD×(+1)+AssD×(+0.5)+C/D×(−1)+SpL×(+1)+IOL×(+0.5)+elC/D×(−1)+TR×(−0.5)+BT×(−0.5)+PMR×(−1)+MMR×(−1)	

Supplementary Table S1. *Cont.*

Category	Quality indicators [1] or Definitions	Numerator	Denominator
Neonatal morbidity and mortality	Apgar score <5 at 5 min	Number of live-born infants with Apgar score <5 at 5 min [2]	Total number of live-born infants (stillborn infants excluded) [2]
	Live-born infants, preterm birth <33 wk	Number of live-born infants with a birth <33 wk [2]	Total number of live-born infants [2]
	Birth weight <5th percentile for gestational age	Number of infants with a birth weight <5th percentile for gestational age [2]	Total number of infants [2]
	Birth weight <2750 g with birth ≥40 wk	Number of infants with a birth weight <2750 g with a birth ≥40 wk [2]	Total number of infants with birth ≥40 wk [2]
	Birth weight <2500 g	Number of infants with a birth weight <2500 g [2]	Total number of infants [2]
	Infants discharged from delivery unit with mother and birth ≥37 wk	Number of live-born infants ≥37 wk discharged from delivery unit with mother [2]	Total number of live-born infants ≥37 wk [2]
	Neonatal transfers in intensive care unit with birth weight >2500 g	Number of live-born infants transferred to a neonatal intensive care unit with a birth weight >2500 g [2]	Total number of live-born infants with birth weight >2500 g (admissions for congenital malformations excluded) [2]
	Neonatal transfers to any neonatal units for inborn infants with birth ≥22 wk	Number of infants transferred to any neonatal unit with a birth ≥22 wk [2]	Total number of live-born infants with a birth ≥22 wk (admissions for congenital malformations excluded) [2]
	Uncomplicated births with a healthy child	Total number of births without any of: cesarean or obstetric intervention, postpartum hemorrhage >1000 mL or perineal laceration, and with a 5-min Apgar score ≥9 [2]	Total number of live-born infants [2]
	Stillbirths ≥28 wk	Number of fetal deaths (including in utero fetal deaths, medical terminations and intrapartum deaths) born ≥28 wk [2]	Total number of births ≥28 wk (live-born or stillborn infants) [2]

Supplementary Table S1. *Cont.*

Category	Quality indicators [1] or Definitions	Numerator	Denominator
Maternal morbidity	Postpartum hemorrhages >500 mL (regardless of mode of delivery)	Number of postpartum hemorrhages >500 mL [2]	Total number of deliveries [2]
	Postpartum hemorrhages >1500 mL (regardless of mode of delivery)	Number of deliveries with a postpartum hemorrhages >1500 mL [2]	Total number of deliveries [2]
	Postpartum hemorrhages >1500 mL for cesareans	Number of postpartum hemorrhages >1500 mL for cesarean [2]	Total number of cesarean deliveries [2]
	Intact perineum in spontaneous delivery	Number of women with an intact perineum (without suture) in spontaneous delivery [2]	Total number of spontaneous vaginal deliveries [2]
	Intact perineum in primiparas	Number of women with an intact perineum (without suture) in primiparas [2]	Total number of deliveries in primiparas [2]
	Perineal lacerations (perineal tears and/or episiotomy)	Number of women with perineal lacerations (tears and/or episiotomy) [2]	Total number of vaginal deliveries [2]
	Perineal lacerations without episiotomy	Number of women with perineal lacerations (tears) without episiotomy [2]	Total number of vaginal deliveries [2]
	First-degree perineal tears	Number of women with 1st-degree perineal tears [4] for vaginal delivery [2]	Total number of vaginal deliveries [2]
	Second degree perineal tears	Number of women with 2nd-degree perineal tears [5] for vaginal delivery [2]	Total number of vaginal deliveries [2]
	Severe perineal tears in vacuum delivery	Number of women with 3rd- and 4th-degree perineal tears [6] for instrumental vaginal delivery using vacuum [2]	Total number of instrumental vaginal deliveries using vacuum [2]
	Adjusted proportion of severe perineal lacerations (third or fourth degree)	Proportion of severe perineal tears (3rd- and 4th-degree) adjusted for maternal age, parity, ethnicity, instrumental extraction, episiotomy, birth weight, and shoulder dystocia	

Wk: weeks of amenorrhea. [1] Quality indicators are expressed as percentages. [2] For births ≥22 wk or birth weight ≥500 g in the absence of a known pregnancy onset. [3] Women with a history of only one child born by cesarean and who have a secondary cesarean for their second delivery. [4] First-degree tears involve damage to vaginal and perineal skin. [5] Second-degree tears involve the posterior vaginal wall and the underlying elevator and perineal muscles. [6] Severe perineal tears include 3rd- and 4th-degree perineal tears. Third-degree tears involve the anal sphincter, with either total or partial damage to the sphincter and fourth-degree tears involving the anal sphincter and tears into the rectal mucosa.
